# Predicting Urban Medical Services Demand in China: An Improved Grey Markov Chain Model by Taylor Approximation

**DOI:** 10.3390/ijerph14080883

**Published:** 2017-08-06

**Authors:** Jinli Duan, Feng Jiao, Qishan Zhang, Zhibin Lin

**Affiliations:** 1 College of Pharmacy, Fujian University of Traditional Chinese Medicine, No. 1 Qiuyang Road, Fuzhou 350122, Fujian, China; ywjoy@126.com; 2 School of Economics and Management, Fuzhou University, No. 2 Xueyuan Road, Fuzhou 350108, Fujian, China; 3 Newcastle University Business School, Newcastle University, 5 Barrack Road, Newcastle upon Tyne NE1 4SE, UK; f.jiao@newcastle.ac.uk; 4 Newcastle Business School, Northumbria University, Newcastle upon Tyne NE1 8ST, UK; Zhibin.lin@northumbria.ac.uk

**Keywords:** medical services demand, Grey Markov chain, Taylor Approximation, prediction

## Abstract

The sharp increase of the aging population has raised the pressure on the current limited medical resources in China. To better allocate resources, a more accurate prediction on medical service demand is very urgently needed. This study aims to improve the prediction on medical services demand in China. To achieve this aim, the study combines Taylor Approximation into the Grey Markov Chain model, and develops a new model named Taylor-Markov Chain GM (1,1) (T-MCGM (1,1)). The new model has been tested by adopting the historical data, which includes the medical service on treatment of diabetes, heart disease, and cerebrovascular disease from 1997 to 2015 in China. The model provides a predication on medical service demand of these three types of disease up to 2022. The results reveal an enormous growth of urban medical service demand in the future. The findings provide practical implications for the Health Administrative Department to allocate medical resources, and help hospitals to manage investments on medical facilities.

## 1. Introduction

The population aged 65 and over has increased almost twice as fast as the younger population in the past two decades [1]. This sharp increase of the aging population has brought enormous pressure to the current medical services system. Moreover, improved living standards, advanced medical science and technology, and higher medical service expectations have further stimulated the demand for medical services [2]. Allocating the limited health care resources has been more challenging than ever before for policy-makers and managers. Developing an accurate and flexible forecasting system for predicting medical services demand thus becomes imperative [3,4].

To predict individual medical services demand, an early study by Grossman [5] applied Becker’s equilibrium analysis framework by integrating a utility function, family production function, income constraint function, and time constraint function. Later studies by Hupert et al. [6], McCarthy et al. [7], Lowthian et al. [8], and Hagihara et al. [9] developed different models based on multiple regression analysis [6,7,8,9,10,11,12,13,14,15]. In addition, there are other forecasting models for predicting medical services demand. For example, the auto regressive moving average model combined with a neural network was established to predict the incidence of scarlet fever, which showed that the forecasting accuracy of the combination model is superior to the simple auto regressive moving average model [16]. A model based on the service goal and trend of the vertebral method was proposed to predict regional medical services demand [17]. The work points method is used to predict annual public health service demand considering gender and age [18]. There are also studies that applied system dynamics simulation model to predict care services demand [19,20].

Among the recent studies, a Markov Chain (MC) model has been widely adopted. For example, Teresa (2012) and Akita (2016) applied the MC model to calculate the transition probability of patients in treatment [21,22]. Ujjwal (2010) presented a Grey-Markov model to forecast energy consumption in India [23]. Chen (2011) presented a Grey Markov model to forecast financial crises for an enterprise [24]. Lin et al. (2013) used a Grey-Markov forecasting model to analyze Taiwan’s IC assembly industry [25]. Kordnoori et al. (2014) applied a Grey Markov model to analyze global ICT development [26]. Edem et al. (2016) proposed a modified Grey-Markov model to predict fire accidents [27]. Peng (2009) proposed a MC model considering the medical service project cost present value to predict medical services demand [28]. Huang used the continuous time homogeneous Markov process to analysis the change of the elderly population health status, to predict long-term care and nursing demand for the over 65-year-old elderly population [29]. Hu (2015) applied the MC model to estimate the elderly health state transition probability, and combined this with the result of population prediction to forecast the demand of elderly and nursing services in different years and different disability states [30]. Argiento et al. (2016) attempted to improve the Bayesian framework by applying the MC model, and developed the patients’ demand evolution for the further prediction [31]. 

The GM (1,1) prediction model was created by Deng (1989), who is the founder of Grey system theory [32]. Many studies in prediction are based on small datasets [33]. Compared with general statistic models, the models based on Grey system theory such as GM (1,1) only need limited data for building and predicting, which could also provide a certain degree of fitting effect to evaluate the reliability [34,35]. Chen (2012) used the Grey GM (1,1) model to predict demand of doctors and beds in Tianjin in 2013–2018 [36]. Xiang (2015) used a multi-factors Grey prediction model for health technical personnel demand forecasting. The prediction accuracy was shown to be better than that of the auto regressive model [37]. Bao (2015) applied GM (1,1) model to predict the disease burden of the aged population in China [38]. Wang (2014) used the GM (1,1) model to predict ischemic heart disease hospitalizations in Shenzhen, China [39].

There are three major limitations in applying these methods. First, the traditional regression analysis requires a large database to guarantee prediction accuracy. Second, applying the MC model requires the calculation of disease transition probability. Disease transaction features vary across different disease categories and patients. The probability matrix of disease transaction is different from one patient to another. Third, the traditional Grey GM (1,1) model is hardly able to provide accurate predication regarding random vibration and high noise. Medical service demand is highly influenced by disease spectrum, living environment, and national health policy. Historic data of medical service demand is limited.

This study aims to address the above limitations in the current medical demand prediction literature by developing a new model which is based on the traditional GM (1,1) model and combines the MC model and Taylor Approximation algorithm. The traditional GM (1,1) model is used to predict the medical services demand. Then the MC model improved by the Taylor Approximation algorithm is implied to estimate the transferring state of the residual sequence in the GM (1,1) model to improve the prediction accuracy. The remainder of the paper is structured as follows: Section 2 describes the procedure of the improving Grey Markov Chain Model by Taylor Approximation. Empirical results for the three types of diseases, including diabetes, heart disease, and cerebrovascular disease are presented in Section 3. Section 4 provides the final comments and conclusions.

## 2. Materials and Methods

The improved T-MCGM (1,1) model is made up of three methods, including GM (1,1), Markov Chain, and Taylor Approximation. The purpose of GM (1,1) is to predict the medical service demand of specific diseases by adopting historic data. That of Markov Chain is to revise the absolute error in GM (1,1) by estimating the transferring state of residual sequence. These two methods combined with Taylor Approximation can effectively search the optimal upper and lower bound parameters for transferring the state of the residual sequence.

### 2.1. The Traditional GM (1,1) Model

The traditional Grey GM (1,1) model is developed as the following procedure:

If we assume the original data series is $X^{\left(0\right)}=\{x^{\left(0\right)}(1),x^{\left(0\right)}(2),...,x^{\left(0\right)}(n)\}$, and $x^{\left(0\right)}(i)>0$, i=1,2,...,n. We can accumulate this data series and achieve a new data series, which is $X^{\left(1\right)}=\{x^{\left(1\right)}(1),x^{\left(1\right)}(2),...,x^{\left(1\right)}(n)\}$, and $x^{\left(1\right)}(j)=\sum_{i=1}^{j}x^{\left(0\right)}(i)$, j=1,2,...,n.

Moreover, the next mean generation sequence is:

$$Z^{1}(n)=\{z^{1}(2),z^{1}(3),...,z^{1}(n)\},\;\mathrm{and}\;z^{1}(k)=\frac{1}{2}[x^{1}(k-1)+x^{1}(k)],k=1,2,...,n.$$

Based on the above the data series, it can develop the whitenization differential equation in the GM (1,1) model as the following:

(1) $$\frac{dx^{\left(1\right)}}{dt}+ax^{\left(1\right)}=b$$

In addition, the difference equation in GM (1,1) is:

(2) $$x^{0}(j)+az^{1}(j)=b$$

In Equations (1) and (2), a is the developing coefficient, and b is the grey action. If assuming $\hat{a}={\left[a,b\right]}^{T}$ as parameter list, and $Y=\left[\begin{array}{c} x^{0}(2)\\ x^{0}(3)\\ ...\\ x^{0}(n) \end{array}\right]$ $B=\left[\begin{array}{cc} -z^{1}(2) & 1\\ -z^{1}(3) & 1\\ ... & ...\\ -z^{1}(n) & 1 \end{array}\right]$, the parameter list can satisfy:

(3) $$\overset{⌢}{a}={\left(a,b\right)}^{T}={\left(B^{T}B\right)}^{-1}B^{T}Y$$

Therefore, it can develop Grey Prediction equation, which is:

(4) $${\hat{x}}^{\left(1\right)}(i+1)=[x^{0}(1)-\frac{b}{a}]e^{ia}+\frac{b}{a}$$

(5) $$x^{\left(0\right)}(i+1)=x^{1}(i+1)-x^{1}(i)=[x^{\left(0\right)}(1)-\frac{b}{a}](1-e^{a})e^{-ai}$$

### 2.2. Grey Markov Chain Prediction Model

Prediction of medical service is highly influenced by random influences, such as a disease’s morbidity and seasonal factors. Thus, the traditional GM (1,1) model has difficulty in predicting accurate medical service demand with original data series. In order to improve the current prediction of medical services demand, the following section applies the Markov Chain model to improve the GM (1,1) model, which is able to predict the transferring of the residual sequence in the GM (1,1) model. The improved model can resolve the randomness and volatility of services demand, and have a more accurate prediction on medical services demands.

#### 2.2.1. The Partition of Transferring

In practice, assuming the actual medical services demands are known, the series of absolute error is obtained between the actual demands and prediction, and it is a non-stationary random series in the Markov chain. In practice, medical service demands are different because of different types of diseases. Thus, this random series can be divided into different statuses, with r denoting one status. Therefore, it can achieve a state transition matrix, which is. In this matrix, each state represents an equidistant margin of error. Each prediction error can be located into the corresponding status. We use $S_{ij}$ to indicate state in residual:

(6) $$S_{ij}\in (L_{ij},U_{ij}),\;j=1,2,...,r$$

In addition, and there are the low bound and upper bound of state in residual, which are calculated as:

(7) $$L_{ij}=\min e(i)+\frac{j-1}{r}[\max e(i)-\min e(i)]$$

(8) $$U_{ij}=\min e(i)+\frac{j}{r}[\max e(i)-\min e(i)]$$

#### 2.2.2. The Establishment of the State Transition Matrix

If we assume that $p_{ij}^{\left(m\right)}$ is the probability of the residual transferring from state i to state j in step *m*. $M_{ij}^{\left(m\right)}$ is the sample size of the residual transferring from state i to state j in step m. $M_{i}$ is the sample size of the residual state i.

Therefore, the Markov state transaction matrix consisted of $p_{ij}^{\left(m\right)}$ can be presented as:

(9) $$P^{\left(m\right)}=\left(\begin{array}{cccc} p_{11}^{\left(m\right)} & p_{12}^{\left(m\right)} & ... & p_{1m}^{\left(m\right)}\\ p_{21}^{\left(m\right)} & p_{22}^{\left(m\right)} & ... & p_{2m}^{\left(m\right)}\\ ... & ... & ... & ...\\ p_{r1}^{\left(m\right)} & p_{r2}^{\left(m\right)} & ... & p_{rr}^{\left(m\right)} \end{array}\right)$$

#### 2.2.3. Prediction of the Grey Markov Chain Model

If we assume $a_{i}(t)$ is the probability of state in period, it can get that $\left\{a_{i}(t),i=1,2,...,r\right\}$ is the state transaction vector in the period. $a_{i}(0)$ will denote the initial probability vector of the state transaction, and it can make use of the initial probability vector and the state transaction matrix, to calculate the probability vector of the predicting objects in any period at any state. This is the model of applying MC to GM (1,1), and the model is presented as follows:

(10) $${\tilde{x}}^{\left(0\right)}(t+1)={\hat{x}}^{\left(0\right)}(t+1)+\sum_{i}^{r}a_{i}(t)⊗v_{i}$$

(11) $$\mathrm{And}\;a_{i}(t)=[a_{1}(t),a_{2}(t),...,a_{r}(t)]=a_{i}(t-1)p^{\left(1\right)}$$

In Equation (10), $⊗v_{i}$ is the grey number exists in a certain residual interval. It can be presented as $⊗v_{i}=(L_{i},U_{i})$, and $L_{i}$ and $U_{i}$ are the lower bound and upper bound of the residual interval respectively [40].

### 2.3. The Development of Grey Markov Chain Model with Taylor Approximation

The new prediction model is based on the traditional MC model, and applies Taylor approximation, to develop the Grey MC prediction model.

In order to develop the Grey MC prediction model, it has to transfer the Grey number. This transaction is named the whitening transformation of the Grey number. In our study, we can whiten this Grey number by following Equation (12):

(12) $$⊗v_{i}=\alpha_{i}L_{i}+(1-\alpha_{i})U_{i}\;i=1,2,...,r$$

In this equation, $\alpha_{i}\in [0,1]$ is the whitening coefficient. By combining this with Equation (11), the Grey Markov Chain model can be developed as follows:

(13) $${\tilde{x}}^{\left(0\right)}(t+1)={\hat{x}}^{\left(0\right)}(t+1)+\sum_{i}^{r}a_{i}(t)[\alpha_{i}L_{i}+(1-\alpha_{i})U_{i}]$$

Based on Equation (13), if we can confirm vectors $\left[\alpha_{1},\alpha_{2},...,\alpha_{r}\right]$, the Grey Markov Chain model can provide the prediction. Hence, the following section will apply Taylor approximation to confirm $\left[\alpha_{1},\alpha_{2},...,\alpha_{r}\right]$ in the minimum convergence tolerance. The solution is provided as follows.

Step 1:  Initialization

Set the number of iterations as K.

Set the initial whitening coefficient as:

(14) $$\alpha_{i}={\left[\alpha_{1},\alpha_{2},...,\alpha_{r}\right]}^{T}={\left[0.5,0.5,...,0.5\right]}^{T}$$

Set the objective function vector as:

(15) $$G={\left[x^{\left(0\right)}(1),x^{\left(0\right)}(2),...,x^{\left(0\right)}(n)\right]}^{T}$$

Set the approximate function vector as:

(16) $$F^{\left(K\right)}={\left[{\tilde{x}}^{\left(0)(K\right)}(1),{\tilde{x}}^{\left(0)(K\right)}(2),...,{\tilde{x}}^{\left(0)(K\right)}(n)\right]}^{T}$$

Set approximate parameter as:

(17) $$\alpha^{\left(K\right)}={\left[\alpha_{1}^{\left(K\right)},\alpha_{2}^{\left(K\right)},...,\alpha_{r}^{\left(K\right)}\right]}^{T}$$

Step 2:  Calculate the approximate function vector $F^{\left(K\right)}$ by applying Taylor series expansion:

(18) $$F^{\left(K+1\right)}=F^{\left(K\right)}+\sum_{i=1}^{r}F_{i}^{\left(K\right)}[\alpha_{i}{}^{\left(K+1\right)}-\alpha_{i}^{\left(K\right)}]$$

(19) $$\begin{array}{l} \begin{array}{c} F_{1}^{\left(K\right)}=\frac{\partial F^{\left(K\right)}}{\partial \alpha_{1}^{\left(K\right)}}\approx \frac{F^{\left(K\right)}[\alpha_{1}^{\left(K\right)}+C_{1}^{\left(K\right)}]-F^{\left(K\right)}\alpha_{1}^{\left(K\right)}}{C_{1}^{\left(K\right)}}\\ F_{2}^{\left(K\right)}=\frac{\partial F^{\left(K\right)}}{\partial \alpha_{2}^{\left(K\right)}}\approx \frac{F^{\left(K\right)}[\alpha_{2}^{\left(K\right)}+C_{2}^{\left(K\right)}]-F^{\left(K\right)}\alpha_{2}^{\left(K\right)}}{C_{2}^{\left(K\right)}}\\ \vdots \\ F_{r}^{\left(K\right)}=\frac{\partial F^{\left(K\right)}}{\partial \alpha_{r}^{\left(K\right)}}\approx \frac{F^{\left(K\right)}[\alpha_{r}^{\left(K\right)}+C_{r}^{\left(K\right)}]-F^{\left(K\right)}\alpha_{r}^{\left(K\right)}}{C_{r}^{\left(K\right)}} \end{array} \end{array}$$

In Equation (20), and:

(20) $$C_{1}^{\left(K\right)}=\frac{\alpha_{1}^{\left(K\right)}}{R_{1}},C_{2}^{\left(K\right)}=\frac{\alpha_{2}^{\left(K\right)}}{R_{2}},...,C_{r}^{\left(k\right)}=\frac{\alpha_{r}^{\left(K\right)}}{R_{r}}$$

where $R_{1},R_{2},...,R_{r}$ is the step length coefficient, which is set as $R_{1}=R_{2},...,R_{r}=200$ in this study.

Step 3:  Set the evaluation function as:

(21) $$Q^{\left(K\right)}={\left[F_{D}^{\left(K\right)}-\sum_{i=1}^{r}F_{i}^{\left(K\right)}\alpha_{i}\right]}^{T}[F_{D}^{\left(K\right)}-\sum_{i=1}^{r}F_{i}^{\left(K\right)}\alpha_{i}]\;\mathrm{where}\;F_{D}^{\left(K\right)}=G-F^{\left(K\right)}$$

(22) $$\eta^{\left(K\right)}=\left[\begin{array}{c} \eta_{1}^{\left(K\right)}\\ \eta_{2}^{\left(K\right)}\\ \vdots \\ \eta_{r}^{\left(K\right)} \end{array}\right]=\left[\begin{array}{c} \alpha_{1}^{\left(K+1\right)}-\alpha_{1}^{\left(K\right)}\\ \alpha_{2}^{\left(K+1\right)}-\alpha_{2}^{\left(K\right)}\\ \vdots \\ \alpha_{r}^{\left(K+1\right)}-\alpha_{r}^{\left(K\right)} \end{array}\right]$$

If we assume a tolerable error ε, at the point which the evaluation function iteration terminates and enters step 4.

Step 4:  Iterate the approximate parameter:

(23) $$Min(Q^{\left(K\right)})\to 0$$

Let:

(24) $$\frac{\partial Q^{\left(K\right)}}{\partial \eta_{1}^{\left(K\right)}}=0,\frac{\partial Q^{\left(K\right)}}{\partial \eta_{2}^{\left(K\right)}}=0,...,\frac{\partial Q^{\left(K\right)}}{\partial \eta_{r}^{\left(K\right)}}=0$$

It can achieve Equation (25).

(25) $$\alpha^{\left(K+1\right)}=\alpha^{\left(K\right)}+{\left[A^{(K)T}A^{\left(K\right)}\right]}^{-1}A^{(K)T}F_{D}^{\left(K\right)},\;\mathrm{and}\;A^{\left(K\right)}=\left[F_{1}^{\left(K\right)},F_{2}^{\left(K\right)},...,F_{r}^{\left(K\right)}\right]$$

Lastly, we need to increase the number of iterations (K = K + 1), and repeat the step 2. When $Q^{\left(K\right)}\leq \varepsilon$, the predicting value of the improved Grey MC model is:

$$F^{\left(K\right)}={\left[{\tilde{x}}^{\left(0)(K\right)}(1),{\tilde{x}}^{\left(0)(K\right)}(2),...,{\tilde{x}}^{\left(0)(K\right)}(n)\right]}^{T}$$

The T-MCGM (1,1) model we developed is unique in three main aspects. First, it has the advantage of creating the prediction without requiring large databases or sample sizes. Second, the Markov Chain model requires the object to be stationary, while the object of T-MCGM (1,1) can be non-stationary. Third, T-MCGM (1,1) is able to revise the volatility and noise in the prediction. Thus, it has a better performance than ARMA and BP in terms of predicated accuracy. To better verify the effectiveness of T-MCGM (1,1), the following section applies it with empirical data, and compares the results with other prediction models.

### 2.4. Data

We used the database of China Health Statistic Yearbook, and obtain the medical services demand of diabetes DD), heart disease (HD) and cerebrovascular disease (CD). These three types of disease are very common in the aging population in China, and they have features that include high incidence rate, multiple causes, and other random factors. Therefore, these diseases can be used as an ideal case to test the improved Grey MC model. Hospitalizing rate within two-weeks is selected as the indicators. The data of hospitalizing rate within two-weeks from 2006 to 2015 are listed in Table 1.

### 2.5. Procedure

**Figure 1:**
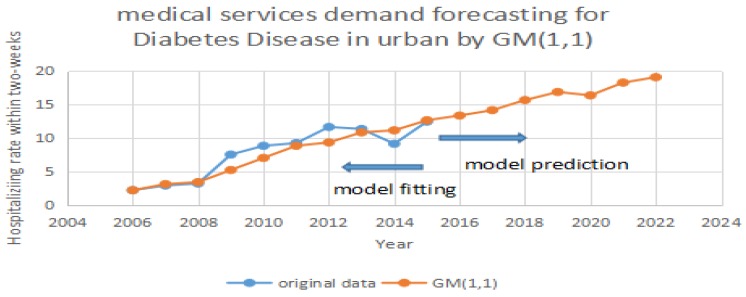
Forecasting diabetes in urban areas by GM (1,1).

**Figure 2:**
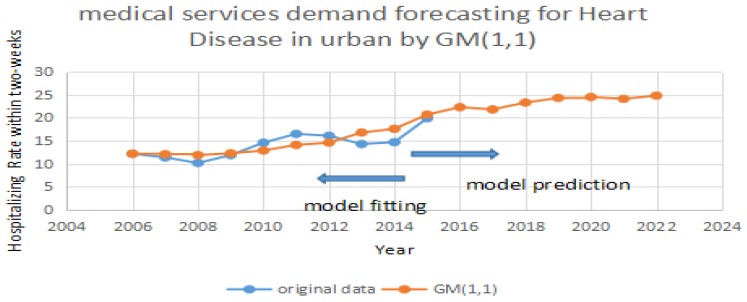
Forecasting for heart disease in urban areas by GM (1,1).

**Figure 3:**
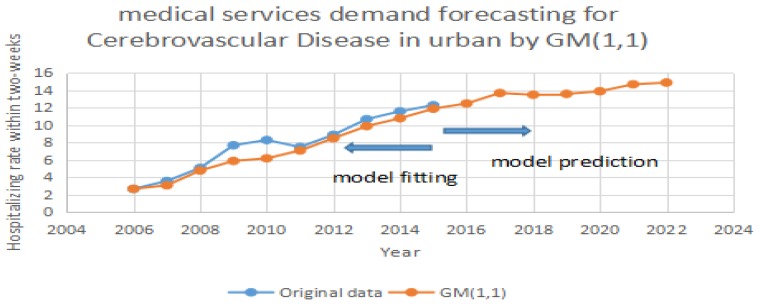
Forecasting cerebrovascular disease in urban areas by GM (1,1).

Based on Table 1, we test the predicting accuracy of the traditional GM (1,1) model. By importing the data in Table 1 into GM (1,1), we can achieve the prediction and errors. In Figure 1, Figure 2 and Figure 3, the blue line represents the original data in Table 1. The red line represents the prediction which is calculated by the traditional GM (1,1) model. It can be seen that the two periods include 2008 to 2013 and 2013 to 2015 have significant errors in Figure 1. It can be seen that two periods including 2007 to 2012 and 2013 to 2015 have significant errors in Figure 2. It can also be seen that one period of 2008 to 2011 has significant errors in Figure 3. Due to the errors, the GM (1,1) model has a weak performance in predicting the medical services demands. Therefore, we purposely apply the same data, and test them using the improved MC model by Taylor Approximation.

**Figure 4:**
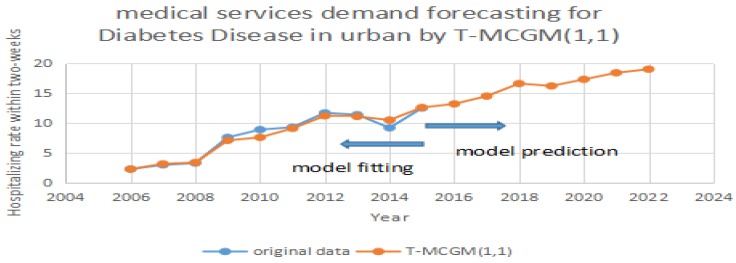
Forecasting diabetes in urban areas by T-MCGM (1,1).

After the model completes the iterations, it can achieve the optimization of the vectors $\left[\alpha_{1},\alpha_{2},...,\alpha_{r}\right]$. By applying Equation (13), it can calculate the prediction of the T-MCGM (1,1) model. In Figure 4, the blue line stands for the original date, the red line represents the prediction of the T-MCGM (1,1) model.

**Figure 5:**
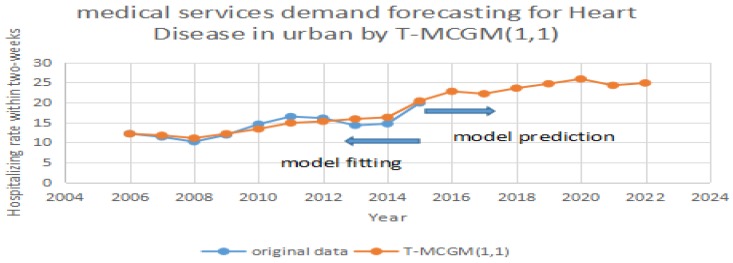
Forecasting heart disease in urban areas by T-MCGM (1,1).

**Figure 6:**
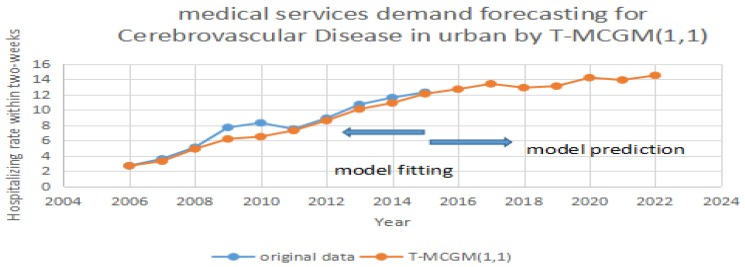
Forecasting cerebrovascular disease in urban areas by T-MCGM (1,1).

Based on Figure 4, Figure 5 and Figure 6, the T-MCGM (1,1) model has performed a better prediction than the traditional GM (1,1) model. Especially for the periods with significant errors by GM (1,1), the prediction of T-MCGM (1,1) is more accurate, and has smaller errors than the traditional GM (1,1) model. Specifically, the prediction precision in Figure 2 by GM (1,1) (MAPE) is 10.3%, and the prediction precision in Figure 5 by T-MCGM (1,1) is 6.23%. From the perspective of long-term prediction, the improvement of the prediction accuracy is acceptable. Moreover, the T-MCGM (1,1) model is based on GM (1,1), and the result by T-MCGM (1,1) is obtained by revising the forecasting result. Therefore, improvement of the prediction accuracy is not significant.

## 3. Results

**Figure 7:**
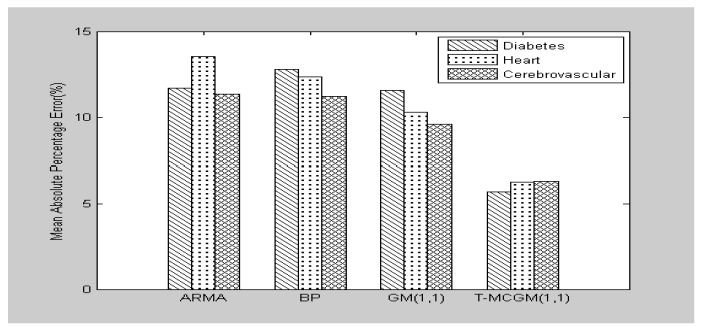
The comparison of fitting results by four models.

In order to test the feasibility of T-MCGM (1,1) model, our study selects other three models which include auto regressive moving average (ARMA), back propagation neural network (BP), and GM (1,1) to compare with T-MCGM (1,1). The test focuses on two criteria, including absolute mean percentage error (MAPE) and root mean square error (RMSE), to compare the four models’ performance in these criteria. The result of the comparison is presented in Figure 7 and Table 2.

As shown in Figure 7 and Table 2, T-MCGM (1,1) has the highest imitation with the original data. Comparing with the traditional GM (1,1) model, T–MCGM (1,1) has reduced 50.95% in MAPE for diabetes. Comparing with BP and ARMA, T-MCGM (1,1) has decreased 55.68% and 51.54% in MAPE for diabetes, respectively. These results are able to prove the feasibility of T-MCGM (1,1) in our study, which has a better prediction on medical services demands than the other three models.

Table 3 shows the forecasting value from 2016 to 2022 for medical services demand by T-MCGM (1,1). For diabetes, there is a significant growth from 2017 to 2018, and from 2018 to 2022, it presents a relatively stable growth. For heart disease, there are two shocks from 2016 to 2017 and from 2020 to 2021. For cerebrovascular disease, there are two periods in slight decline from 2016 to 2017, and from 2021 to 2022.

## 4. Discussion

By applying the T-MCGM (1,1) model in urban areas of China, we have noticed that there will be an increase of the demand of medical services from 2016 to 2022. Regarding the types of disease discussed above, some suggestions are provided to be considered in further medical resource allocation.

The demand for medical services in diabetes and cerebrovascular disease has faster increasing rates than heart disease. This can be explained as individuals older than 65 are the main patients of the first two. Different diseases require different medical resources. For example, the patients with chronic disease need plenty of drug resources and nursing staff, while patients with serious acute disease need surgical treatment. Therefore, medical service prediction should consider the different types of disease.

There is a larger fitting error in the demand fitting curve in 2010–2011. This may be caused by the New Medical Reform Policy which has been practiced since 2009. The scope of urban medical insurance is expanding and the proportion of reimbursement is increasing. The demand of medical services for chronic disease is stimulated. The prediction models based on GM (1,1) are weak, as without considering policy-driving factors, the demand increase of the prediction for medical services presents the hysteresis characteristics.

The individual living standards and individual health awareness have increased in many developing countries in recent years. Therefore, the T-MCGM (1,1) model for medical services prediction can be applied to these countries for releasing their limited medical resources, such as India and Brazil. It is especially able to contribute a more accurate medical demand prediction in these countries, and provide adequate resources for medical service provision.

The prediction of the demand of medical services for specific diseases has practical implications for the medical and health authorities, hospitals, medical colleges, and drug manufacturers. Different diseases require different medical resources. For example, the patients with chronic disease need plenty of drug resources and nursing staff, while patients with serious acute disease need surgical treatment and beds in hospitals. In this paper, the forecasting results from 2016 to 2022 can promote health service organizations to allocate limited medical resources, including doctors, nurses, pharmacists, and other human resources and drug resources. However, due to the limited access to the administration data on medical resources allocation, the practice of optimizing limited medical resources has not been discussed from a technical perspective in this paper. This aspect needs to be simulated and studied in further research.

Generally, T-MCGM (1,1) overcomes the shortage of omitting the driving factors in the GM (1,1) model, and also weakens the volatility and noisiness of data by combining the Markov chain. Regarding this advantage, T-MCGM (1,1) has presented good adaptability for poor data and small samples. With using the same group of historical data of medical service demands, the model has a better prediction than the ARMA and BP models. In terms of the volatility and noisy of databases in medical service demands, the T-MCGM (1,1) model is more suitable for predicting the demands of patients.

However, the development of T-MCGM (1,1) requires combining the three different models, which are based on a large amount of calculation and time consumption. Thus, it is not easy to carry out for solving urgent predictions of medical service demands and allocations of health resources in reality. Further research includes considering the prediction on rural areas in China, and focusing on comparing the difference between urban areas and rural areas in medical services demand. In this regard, the further research aims to contribute more allocating schemes for urban and rural areas.

## 5. Conclusions

The T-MCGM (1,1) model represents a feasible and efficient method for predicting the trend of annual medical services demand for various diseases in China. The prediction results can be used by the national health administration to allocate limited medical resources, and for develop hospitals and other health organizations to develop proactive service plans.

## Figures and Tables

**Table 1:** Original data from 2006 to 2015.

Year	2006	2007	2008	2009	2010	2011	2012	2013	2014	2015
DD	2.3	3	3.3	7.6	8.2	9.3	10.7	11.4	10.5	12.5
HD	12.2	11.4	10.2	11.9	13.5	14.7	16.1	17.5	18.3	19.9
CD	2.7	6.7	3.6	5.1	6.9	7.5	8.9	10.7	11.6	12.3

**Table 2:** The prediction precision by four various models.

Model	Diabetes Disease		Heart Disease		Cerebrovascular	
	MAPE	RMSE	MAPE	RMSE	MAPE	RMSE
ARMA	11.68%	0.5427	13.53%	0.7011	11.32%	0.4936
BP	12.77%	0.6481	12.35%	0.5673	11.21%	0.4922
GM (1,1)	11.54%	0.4284	10.30%	0.5453	9.59%	0.3254
T-MCGM (1,1)	5.66%	0.2016	6.23%	0.3333	6.31%	0.2577

**Table 3:** The prediction results for three types of disease by T-MCGM (1,1) (‰).

Year	2016	2017	2018	2019	2020	2021	2022
DD	13.2	14.5	16.6	16.2	17.3	18.4	19
HD	22.8	22.2	23.6	24.7	25.9	24.3	24.9
CD	12.7	13.4	12.9	13.1	14.2	13.9	14.5
